# Early branching arbuscular mycorrhizal fungus *Paraglomus occultum* carries a small and repeat-poor genome compared to relatives in the Glomeromycotina

**DOI:** 10.1099/mgen.0.000810

**Published:** 2022-04-22

**Authors:** Mathu Malar C, Yan Wang, Jason E. Stajich, Vasilis Kokkoris, Matthew Villeneuve-Laroche, Gokalp Yildirir, Nicolas Corradi

**Affiliations:** ^1^​ Department of Biology, University of Ottawa, Ottawa, ON K1N 6N5, Canada; ^2^​ Department of Biological Sciences, University of Toronto Scarborough, Toronto, Canada; ^3^​ Department of Ecology and Evolutionary Biology, University of Toronto, Toronto, Canada; ^4^​ Department of Microbiology and Plant Pathology, University of California–Riverside, Riverside, CA, USA; ^5^​ Institute for Integrative Genome Biology, University of California–Riverside, Riverside, CA, USA

**Keywords:** AMF, *Paraglomus occultum*, early diverging

## Abstract

The arbuscular mycorrhizal fungi (AMFs) are obligate root symbionts in the subphylum Glomeromycotina that can benefit land plants by increasing their soil nutrient uptake in exchange for photosynthetically fixed carbon sources. To date, annotated genome data from representatives of the AMF orders Glomerales, Diversisporales and Archaeosporales have shown that these organisms have large and highly repeated genomes, and no genes to produce sugars and fatty acids. This led to the hypothesis that the most recent common ancestor (MRCA) of Glomeromycotina was fully dependent on plants for nutrition. Here, we aimed to further test this hypothesis by obtaining annotated genome data from a member of the early diverging order Paraglomerales (*Paraglomus occultum*). Genome analyses showed this species carries a 39.6 Mb genome and considerably fewer genes and repeats compared to most AMF relatives with annotated genomes. Consistent with phylogenies based on ribosomal genes, our phylogenetic analyses suggest *P. occultum* as the earliest diverged branch within Glomeromycotina. Overall, our analyses support the view that the MRCA of Glomeromycotina carried hallmarks of obligate plant biotrophy. The small genome size and content of *P. occultum* could either reflect adaptive reductive processes affecting some early AMF lineages, or indicate that the high gene and repeat family diversity thought to drive AMF adaptability to host and environmental change was not an ancestral feature of these prominent plant symbionts.

## Data Summary

**Impact Statement**

Most plants undergo underground symbioses with single celled organisms called arbuscular mycorrhizal fungi (AMF). This symbiosis is prominent in terrestrial ecosystems, where AMFsprovide higher access to soil nutrients to plants and represent a food source for multiple organisms. To date, genome data has shown that these fungi carry relatively large and highly repeated genomes, and genome comparisons revealed that AMF have lost the ability to produce sugars and lipids; meaning that these are fully depend on plants for nutrition. In this paper, we aimed to support these findings by sequencing the genome of the early AMF lineage *Paraglomus occultum*. Our analyses confirm that all AMF sequenced to date cannot produce lipids and sugars. However, we found that *P. occultum* carries a very small and gene-poor genome compared to its AMF relatives. This information drastically changes our view that all AMF genomes evolved to be large and repeat rich as a means to improve their adaptability to change.

All genome data is available from the National Center for Biotechnology Information BioProject PRJNA767608 and from Zenodo (https://doi.org/10.5281/zenodo.5879318).

## Introduction

Arbuscular mycorrhizal fungi (AMF) (subphylum Glomeromycotina) [1–3] form symbiotic relationships with over 70 % of vascular land plants [4, 5]. The arbuscular mycorrhizal symbiosis (AMS) emerged over 400 million years ago [6], and plays a major role in extant terrestrial ecosystems by allowing plant roots to efficiently acquire poorly soluble soil nutrients (phosphate, nitrogen and other trace elements) in exchange for carbon sources [4]. During AMS, the fungal partner colonizes the plant roots to produce tree-like structures (arbuscules) that promote bidirectional nutrient transfers between partners, including carbohydrates and lipids from plants that AMFs utilize as an energy resource [7]. AMF also interact with the soil microbiome and play a major role in ecosystems and biodiversity by enhancing plant resistance against pathogens, as well as abiotic and biotic stress [8, 9].

AMF produce hyphae and spores that harbour thousands of nuclei within one cytoplasm, and studies on model species have revealed that these organisms are either homokaryotic (all coexisting nuclei carry one dominant genotype) or heterokaryotic (nuclei derive from two parental strains) [10, 11]. Genome analyses have also shown that these organisms have lost genes involved in the production of sugars and fatty acids [12–15], and it was recently proposed that these were lost in the most recent common ancestor (MRCA) of this subphylum [16].

Currently, the Glomeromycotina are subdivided into four orders – i.e. Glomerales, Diversisporales, Archaeosporales and Paraglomerales [17]. Among these, species in the Paraglomerales are understudied and currently the only ones without a publicly available annotated genome. The lack of information on the gene repertoires from Paraglomerales primarily results from difficulties in extracting contaminant-free DNA from pot-cultured representatives of this order. To circumvent this, studies have either used poly-A tailed RNA [5] or single nucleus sequencing [18] as a means to remove obvious bacterial and other non-AMF contaminants. While these approaches produced sequence data of sufficient quality to perform phylogenetic analyses, their high fragmentation combined with the absence of gene annotation made these datasets unsuitable for comprehensive comparative genome studies.

Species in the Paraglomerales inhabit most terrestrial environments where other AMF families co-exist. In communities studies, members of this order vary in abundance compared to other AMF taxa, depending on the soil conditions and geographical location [19–22]. There is also evidence that more intensive (disruptive) agricultural practices have direct negative impacts on the abundance of Paraglomerales compared to other species [23, 24], and that species in this order may be less beneficial in stimulating both plant growth and nutrient uptake by roots compared to representatives of the Glomerales and Diversisporales [25]. However, whether any of these traits derive from genetic or genomic traits unique to this AMF order is currently unknown.

To tackle this question, we obtained an annotated draft genome of *Paraglomus occultum* from less than 100 ng genomic DNA isolated from pot-cultured spores using a *k*-mer based [26] metagenomics approach. Our phylogenetic and comparative analyses revealed that this species carries a small and streamlined genome size and content compared to Glomeromycotina relatives, and suggest that Paraglomerales could represent the earliest phylogenetic node of this group of prominent symbionts.

## Methods

### Cultivation of *P. occultum* samples

Quiescent spores from *P. occultum* (DAOM 240472) were harvested from pot-cultures available at the Glomeromycotina *in vivo* pot-culture collection at Agriculture and Agri-Food Canada (AAFC) (Ottawa, ON, Canada). This fungal collection is regularly checked for contamination using both morphological and molecular techniques (i.e. small subunit of the rRNA gene, SSU; H+ATPases). Using an inverted microscope, only quiescent spores showing viable morphology and lipid contents were used for downstream DNA extraction. Spores were collected with a sieve cascade of descending size ranging from 500 to 63 mm, and the lowest fraction was then placed in a 50 ml Falcon tube with the addition of 50 % (w/w) sucrose solution. Following centrifugation at 5000 ***g*** for 5 min, single spores were isolated from the supernatant, and general species identification was confirmed using morphological descriptions as detailed by Beaudet *et al.* [5]. Spores were surface sterilized using successive baths of chloramine-T, 2 % Tween 20 for 2 min, and sterile distilled water for 1 min, then incubated overnight in a solution of 0.2 mg streptomycin ml^−1^ and 0.1 mg gentamicin ml^−1^, and washed in a final bath of sterile distilled water. Sterilized spores were stored at 4 °C.

### DNA extraction and genome sequencing and assembly

DNA was extracted from 250 individual spores of *P. occultum* using the Qiagen DNA extraction kit, following the manufacturer’s procedures. This resulted in the extraction of 2 ng DNA. The DNA was sent to Fasteris to generate Nextera Illumina paired-end libraries, which were sequenced using the HiSeq 2500 platform. This procedure generated over 12 billion base pairs of sequence data with 93 % of base pairs exceeding a quality score greater than or equal to 30 (Q30). Poor quality and adapter sequences were trimmed using Trimmomatic with the following parameters of ILLUMINACLIP: 2 : 30 : 10 SLIDINGWINDOW: 5 : 20 LEADING:5 TRAILING:5 MINLEN:50.

The resulting reads were assembled using metaSPAdes v3.11.1 [27], and assembled contigs were binned based on tetra-nucleotide signatures using concoct [28], as described previously [16]. Binned clusters were annotated using the blast v 2.6.0+ [29, 30] nr database with *E* value cut-off 1×10^−5^, and clusters matching to bacteria were removed. The retained AMF contigs were used as a reference database to filter the total paired-end reads with bwa [31] using default parameters, where reads mapping to the filtered contigs were extracted to construct a cleaned paired-end sequence library. These cleaned libraries were *de novo* assembled with SPAdes v3.11.1 [32] with parameters of -k 21,33,55,77 --careful. An additional round of tblastn searches of the SPAdes assembled contigs against the National Center for Biotechnology Information (NCBI) nr database was performed to remove detected further bacterial contamination. K-mer (*k*=21) was used on cleaned and filtered reads to estimate the genome size of *P. occultum* using jellyfish 1.1.12 [33].

### Genome annotation

The assembled genome was soft masked before gene prediction using tantan [34], and genome annotation was performed using Funannotate v1.7.4 (https://funannotate.readthedocs.io/) [https://doi.org/10.5281/zenodo.3679386]. Gene annotation was carried out using published *Paraglomus* RNA transcripts and RNA-seq data [5]. Gene functions were identified using Diamond blastx [30], Pfam domain analysis was performed using Pfamscan [35, 36]. Carbohydrate-active enzymes (CAZymes) were identified using the dbCAN2 server [37, 38] and secretory proteins using published pipelines [14, 37]. Metabolic pathway analysis was performed using the KAAS KEGG server [39], and eggNOG 5.0 [40] was used to classify the orthologue gene functions of Mortierellomycotina and Glomeromycotina genomes used in this study. Transposable elements were predicted using TransposonPSI [41]. The completeness of the genome assembly was assessed with busco version 4.0 [42] on protein datasets with default parameters using the fungal gene dataset [fungi_odb10].

### Protein orthology analyses

For comparative analyses, published genomes from Glomeromycotina, Mortierellomycotina and Mucoromycotina (10 species in total) were downloaded from the Joint Genome Institute (JGI) portal MycoCosm database [43, 44], and the predicted proteins were clustered with fastortho using 50 % identify and coverage [14]. From the clustered orthogroup, orthologues were classified into conserved genes (proteins shared by all genomes), dispensable genes (proteins that are present in at least two genomes), and species-specific genes (taxonomically restricted proteins). For each category, we also identified duplicated genes.

Single copy core genes were aligned using mafft [45] with default parameters and the resulting alignments were trimmed to remove positions with gaps using trimAl v1.4. rev22 [46]. A maximum-likelihood tree was reconstructed using iq-tree v1.6.12 [47] with 10 000 replicates of SH-aLRT. Trees were visualized and annotated with FigTree v1.4.4 (https://beast.community/figtree).

### Phylogenetic analysis and alternative topology test

Phylogenomic analysis was carried out using the ‘fungi_odb10’ dataset included in the busco v4.0 package. Profile hidden-Markov-models of these markers were used to identify homologues in *P. occultum* and an additional 44 fungal genomes using hmmer3 (v3.1b2) employed in the PHYling pipeline (https://doi.org/10.5281/zenodo.1257002). A total of 603 conserved and single-copy proteins were identified, aligned and concatenated for the following phylogenetic analyses. The phylogenetic tree was reconstructed using the maximum-likelihood approach with the best-fit substitution model option for individual partitions defined by each gene marker implemented in iq-tree v.1.6. The final analysis was performed on 195 323 sites with 170 865 distinct patterns contributing to the tree structures.

**Fig. 1.**
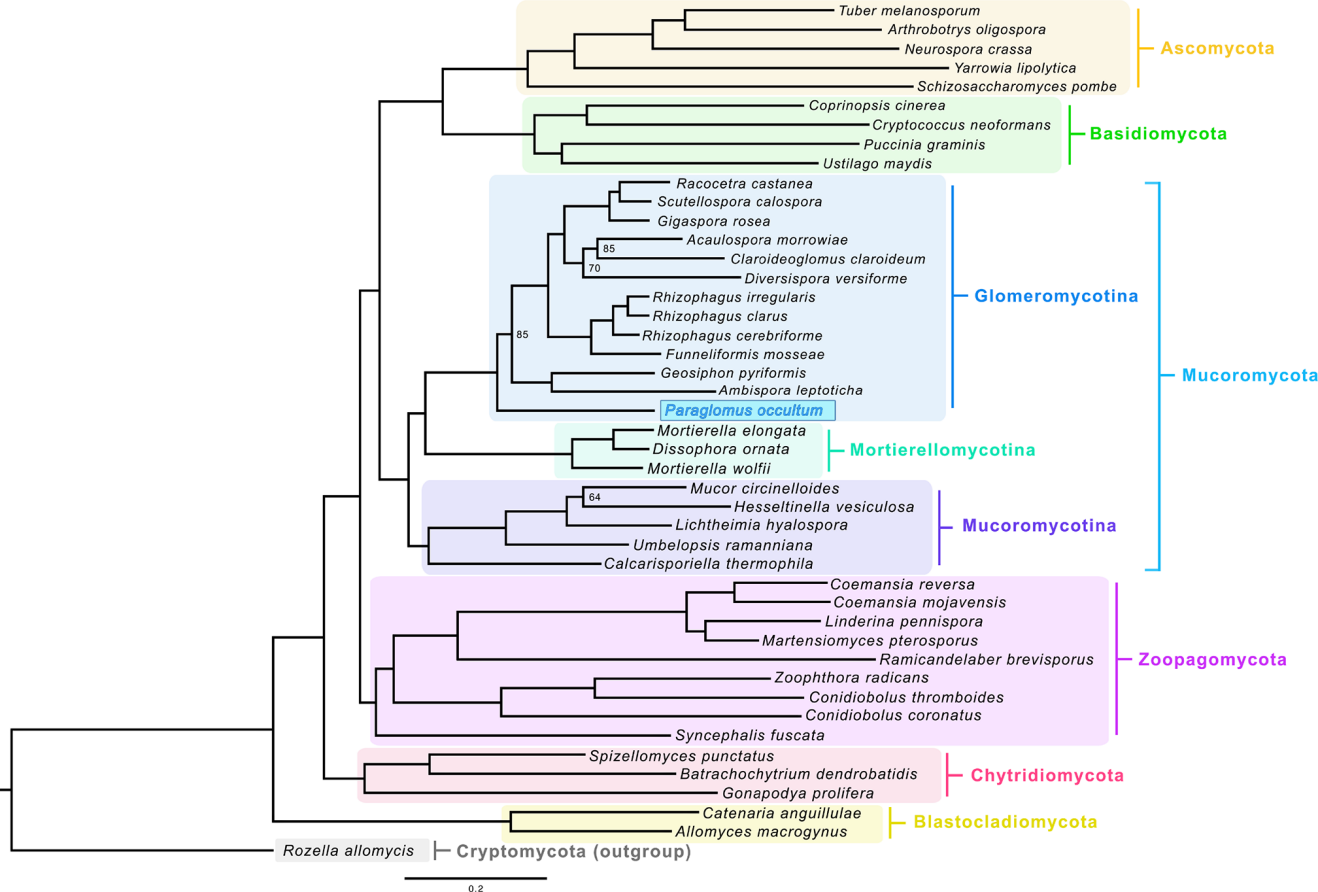
Phylogenetic tree showing the placement of *P. occultum* in the Glomeromycotina clade. The tree was resolved using iq-tree on a concatenated alignment of 434 protein-encoding genes. Numbers indicate nodes with less than 95 % bootstrap support. Branches are coloured according to their phylum.The scale bar indicates number of substitutions per site.

To test the likelihood of alternative phylogenetic placements of *P. occultum*, we conducted constraint tree searches using partially forced tree topology files (Alt_T1, Alt_T2 and Alt_T3 as illustrated in Fig. S1, available with the online version of this article) with the ‘-g’ option implemented in the iq-tree package. Tree files of the best topology (as shown in Fig. 1) and the alternative ones (Fig. S1) were included in the computational analysis for log-likelihoods using Kishino−Hasegawa test, Shimodaira−Hasegawa test, expected likelihood weight, and approximately unbiased test via the ‘-zb’ and ‘-au’ parameters included in the iq-tree package [48–52]. Reliable results were achieved using 10 000 resampling estimated log-likelihood (RELL) replicates.

## Results

### Genomic characteristics of *P. occultum*

DNA isolated from *P. occultum* spores was sequenced using two Illumina libraries with read lengths of 125 bp, producing a total of 54 million paired-end reads. The genome was assembled using the *k*-mer binning based approach recently used to acquire the genome of *Geosiphon pyriformis* [16] and *Diversispora epigea* [13]. This analysis generated 4722 scaffolds for *P. occultum* with a total length of 44 Mb, which compares to sizes of 140 Mb and above for publicly available annotated genomes. In total, 10 145 genes were predicted in the genome, which compares to a mean of 24 000 genes for other AMF taxa, and the *P. occultum* gene density and G+C content is significantly higher than other AMFs (Table 1).

**Table 1.** Genome assembly statistics of *P. occultum* with other Mucoromycota genomes used in this study

Genome	Assembly size (Mb)	No. of scaffolds	Scaffold N50	Largest scaffold (kb)	Repeat %	busco completeness % using V4	G+C (mol%)	Gene density per 10 kb
*Paraglomus occultum*	44	4722	160	104.458	12.78	92.2	36.77	2.276
*Geosiphon pyriformis*	129	795	703	2733.91	64.35	93.2	29.25	1.893
*Gigaspora rosea* V1.0	597.95	7526	734	1204.75	63.44	96.3	28.81	0.550
*Rhizophagus cerebriforme* DAOM227022 V1.0	136.89	2592	266	709.02	24.77	97.2	26.55	1.574
*Rhizophagus irregularis* DAOM 197198V2.0	136.80	1123	129	1375.86	26.38	96.6	27.53	1.913
*Diversispora versiformis* strain IT104	147	731	434	2010.39	43.6	95.5	25.1	1.810
*Rhizopus microsporus* ATCC 11559 V1	25.97	131	8	2782.17	4.68	95.7	37.48	4.610
*Mucor circinelloides* CBS 277.49 V2.0	36.59	26	4	6050.25	20.38	95.3	42.17	3.194
*Phycomyces blakesleenus* NRRL1555 V2.0	53.94	80	11	4452.46	9.74	95.7	35.78	3.062
*Mortierella elongata* AG-77	49.86	473	31	1526.29	4.63	99	48.05	3.002

Overall, the *P. occultum* genome size and gene counts are approximately a third of those found in other AMF. While this small size may suggest that a large portion of the *P. occultum* genome was missed/lost during the assembly process, busco completeness analyses were virtually identical to those found in other sequenced non-model AMF genomes (92.2 %; Table 1) [13–16]. The reduced genome size and repeat content we observed is also validated by assembly sizes obtained by others on the same species and *Paraglomus brasilianum* using independent approaches – i.e. single nucleus sequencing (49 Mb in the work by Montoliu-Nerin *et al*. [18] vs 44 Mb for this study).

**Fig. 2.**
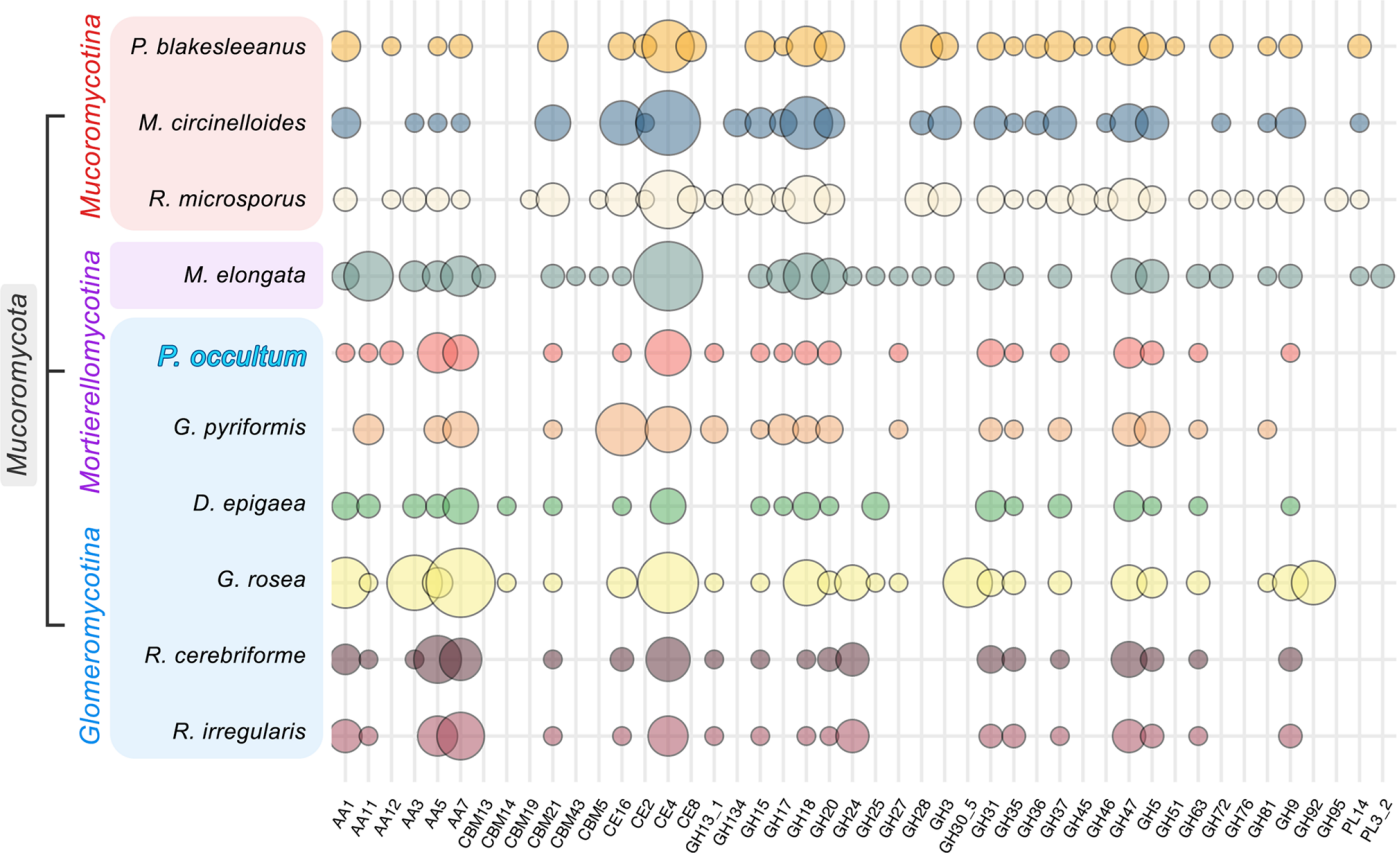
Plot showing the count, presence and absence of plant cell wall degrading enzyme (PCWDE) categories. The *x*-axis shows the categories of PCWDE-encoding enzymes and the *y*-axis shows the list of Mucoromycota species used in this genomics study. Species highlighted in orange represent Mucoromycotina, purple highlighted species are from Mortierellomycotina, and the blue colour represents the species from the Glomeromycotina clade.

Our secretome analysis revealed the presence of 209 putative secreted proteins (Table S1) (Supplementary Material 1) and 120 CAZymes in this species (File S1). The genome also contains 52 plant cell wall degrading enzymes, on a par with numbers found in other AMF species (Fig. 2, File S2). Like all sequenced AMFs, *P. occultum* lacks the ‘Missing Glomeromycotina Core Genes’ (MGCGs), including those involved in the production of fatty acids and sugars (File S3). Our data also indicates this species is likely haploid and homokaryotic – i.e. mapping reads onto the *P. occultum* genome does not reveal evidence of two distinct genotypes [10, 53, 54]. The genome also carries a complete set of meiosis-specific genes (Table S2), further supporting the presence of cryptic (para)sexual reproduction across the subphylum [55]. Analyses of metabolic pathways show that *P. occultum* carries genes involved in nitrogen metabolism and associated transporters in numbers similar to those found in AMF relatives (File S4).

### Phylogenetic placement of *P. occultum*

A set of 603 conserved single-copy genes that includes those from *P. occultum* was used to reconstruct a phylogenetic tree of the fungal kingdom (Fig. 1). These analyses fully support the monophyly of Glomeromycotina and its close relationship with Mortierellomycotina within the phylum Mucoromycota [3, 56]. Remarkably, in support of rRNA-based phylogenies [57, 58], but in minor disagreement with recent protein-code-based work [5, 16, 18], our analyses place *P. occultum* as the earliest lineage within the Glomeromycotina phylogeny [5, 16, 18]. Furthermore, our analyses place *Claroideoglomus claroideum* (*Claroideoglomus* is a genus that is notoriously difficult to assess phylogenetically) within Diversisporales, while other studies placed this genus either as a sister clade to Diversisporales/Glomerales [16, 18] or as a member of Glomerales [57, 58].

The placement of *P. occultum* in our analyses has full statistical support. Alternative topologies that forced this species as a sister lineage to the *Geosiphon–Ambispora* clade (Alt-T1; Fig. S1) or as being associated with Glomerales or Diversisporales (Alt-T2, Alt-T3; Fig. S1) were either rejected (Alt_T2 and Alt_T3) or less favoured (Alt_T1) using statistical tests implemented in iq-tree, including the KH, SH, ELW and AU tests (File S5).

### Shared and unique gene and genome characteristics of *P. occultum*

**Fig. 3.**
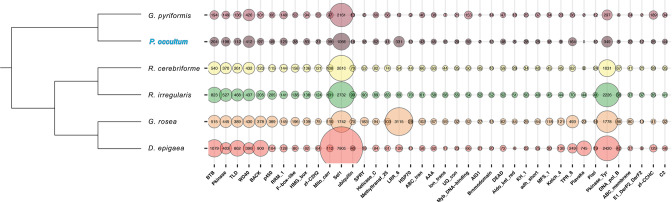
Phylogenetic tree with bubble plot showing the comparison of all the top 40 Pfam domains of AMF genomes used in this study. The *x*-axis represents Pfam functional domains and the *y*-axis represents the species of Glomeromycotina genomes.

Glomeromycotina genomes are expected to be large relative to most fungi, and carry large gene family expansions [11, 12, 14, 59]. *P. occultum* carries gene expansions of the tetratricopeptide repeat Sel1-, the homodimerization BTB (broad-complex, tramtrack and bric-a-brac) and WD-40 domain-containing protein domains (Fig. 3, File S6). However, in this species, most of these domains are less abundant compared to other AMF taxa with annotated genomes. For example, *P. occultum* carries 1066 Sel1 and 349 Pkinase Tyr motifs, compared to a mean of 3400 and 1500 for other Glomeromycotina with sequenced genomes (Fig. 3, File S6). Despite its relatively smaller number of protein domains, the *P. occultum* genome shows enrichment in My-DNA binding domains and zinc-finger motifs.

**Fig. 4.**
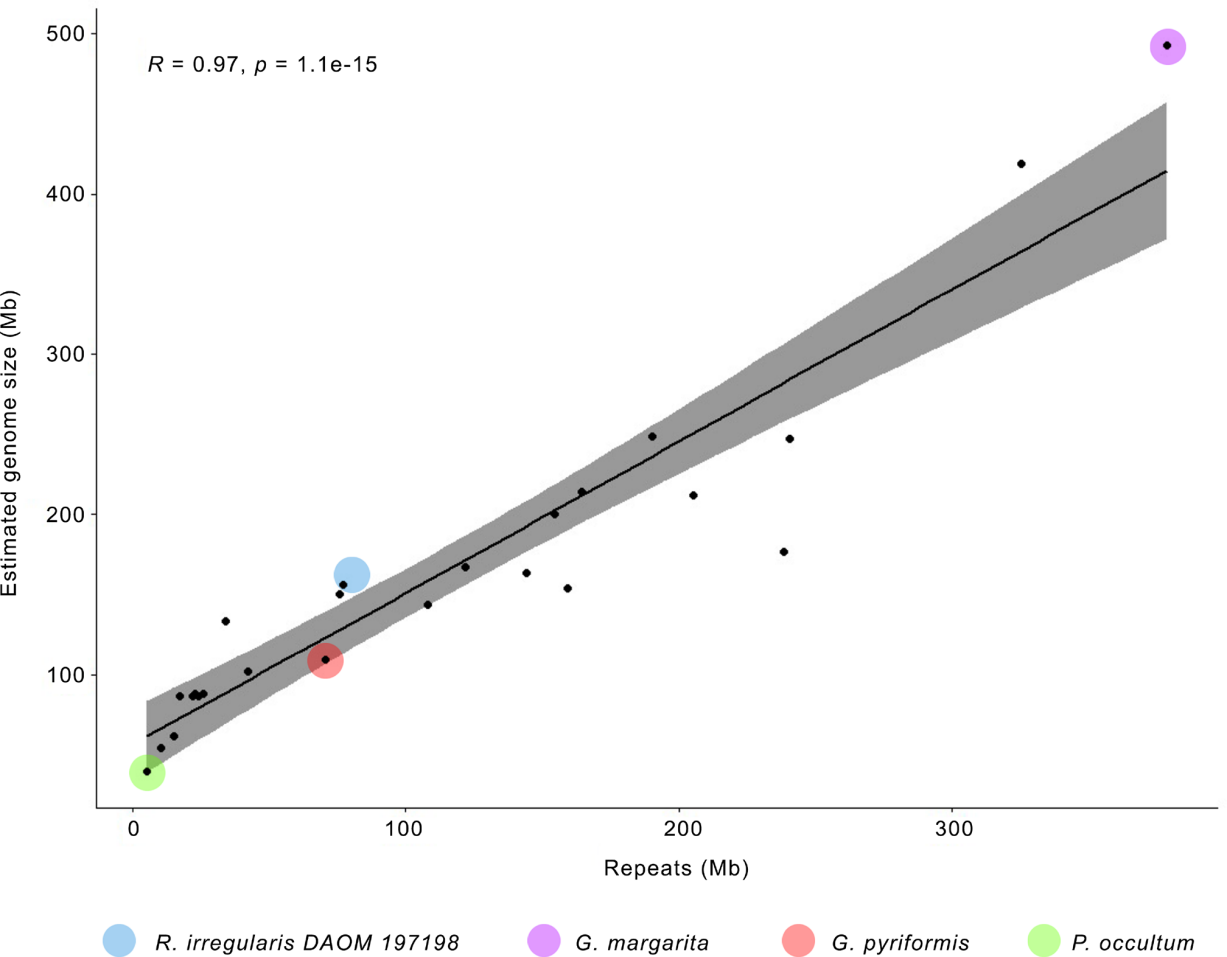
Relationship between repeat content and estimated genome size. All AMF species from Merce *et al.* (2021) [18] are used here along the assembled genome of *P. occultum*. The blue circle represents *Rhizophagus irregularis* and the purple circle represents *Gigaspora margarita* from Merce *et al.* (2021) [18]; the red circle represents *Geosiphon pyriformis* from *Malar C et al.* (2021) [16], and the green circle represent the *P. occultum* genome from this study.

*P. occultum* also contains a fraction of transposable elements found in Glomeromycotina relatives – e.g. 882 (or 0.32 per Mb) transposable element families compared to a mean of 16400 (or 10.17 per Mb) for other Glomeromycotina. These numbers are similar (or higher) to those found in members of Mucoromycotina and Mortierellomycotina with similar genome sizes (Fig. S2, File S7). By comparing our data with genome size and repeat estimates recently obtained from other early AMF taxa, including another member of Paraglomerales (*P. brasilianum*) [18], we found that genome size in AMF significantly correlates with repeat content in the Glomeromycotina (Fig. 4).

**Fig. 5.**
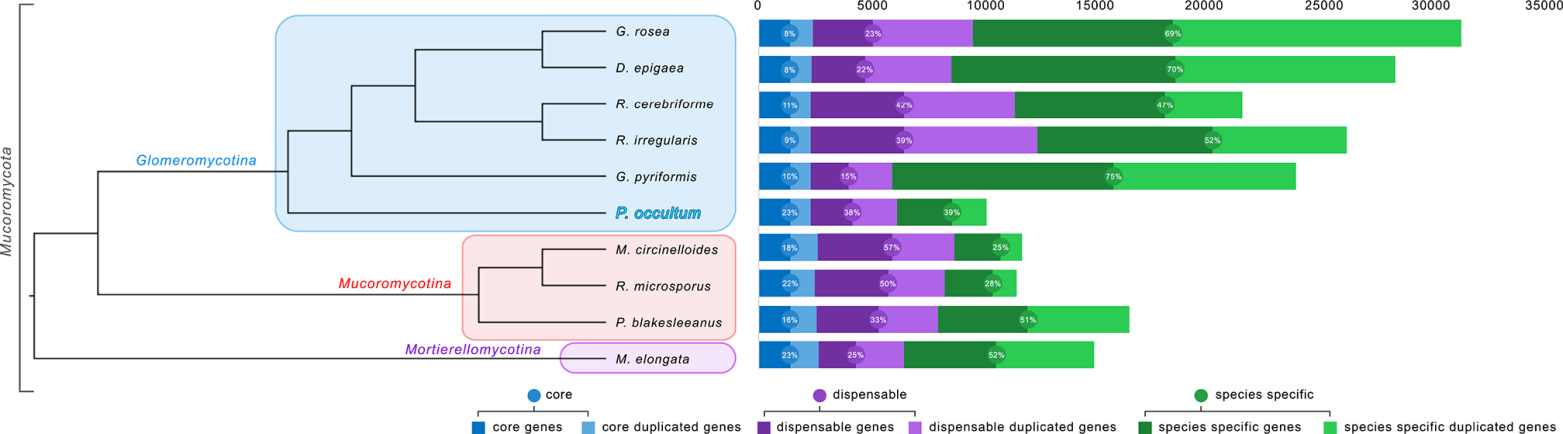
Organismal phylogeny with bar graphs representing conserved proteins shared among the ten species: core proteins (dark blue colour), duplicated core proteins (light blue colour), dispensable proteins (dark purple colour), duplicated dispensable proteins (light purple colour), species-specific proteins (dark green colour), duplicated species-specific proteins (light green colour).

To identify core and unique Glomeromycotina genes, we clustered the predicted protein families of six Glomeromycotina and four Mucoromycotina genomes to detect orthologous groups. Consistent with recent studies [14], orthologues can be classified into core genes (shared among all Mucoromycota), dispensable genes (shared by at least two species of Mucoromycotina or Glomeromycotina species), and species-specific genes (Fig. 5). Due to its small genome size, *P. occultum* contains more than double the percentage of core genes (23 %, compared to ~10 % for other Glomeromycotina) and, on a par with values found in Mortierellomycotina, significantly less species-specific and dispensable genes (39 % compared to ~50 %) (Fig. 5). *P. occultum* also has 200 gene orthologue losses. involved in a variety of putative functions (File S8).

## Discussion

### Paraglomerales may represent the earliest AMF phylogenetic node

To date, AMF taxonomy is primarily generated by linking a small number of morphological traits (e.g. spore structure and content) with phylogenetic analyses of the small subunit of the rRNA operon [17, 60]. These rRNA-based analyses supported the existence of four AMF orders – Diversisporales, Glomerales, Archaeosporales and Paraglomerales – and in these the Paraglomerales represent the earliest lineage [61]. In recent years, the advent of new sequencing methods generated protein-encoding sequence data from poorly studied lineages, and its use in phylogenomics produced a different phylogenetic scenario in which the Paraglomerales group is in a clade that contains members of the Archaeosporales (*Ambispora* and *Geosiphon*) [5, 18, 23, 24].

In this study, an extended orthologue gene set extracted from the *P. occultum* genome produced phylogenies surprisingly consistent with those obtained using rRNA sequences. However, we were also unable to reject an alternative placement of *P. occultum* with members of the Archaeosporales. As such, both current placements of Paraglomerales within the Glomeromycotina phylogeny – i.e. earliest node, or with members of Archaeospora – are equally supported by current phylogenetic analyses, and the acquisition and analysis of annotated genome data from early diverging AMF species will hopefully clarify those relationships.

### A streamlined genome in the MRCA of all extant Glomeromycotina or lineage-specific gene losses?

The present work showed that the *P. occultum* genome has a size, and gene and repeat counts more similar to members of the Mortierellomycotina – i.e. the sister clade of Glomeromycotina – than to most AMF species. These findings are supported by busco values indicating that the *P. occultum* genome space is largely sampled, and by reports of virtually identical results obtained on two members of the Paraglomerales (*P. occultum* and *P. brasilianum*) using independent approaches [18].

Those genome features, combined with the early placement of *P. occultum* in the phylogeny, suggest two contrasting scenarios for Glomeromycotina genome evolution. On one hand, the streamlined features of the *P. occultum* genome could indicate that the MRCA of Glomeromycotina was an obligate plant biotroph but also gene and repeat poor. If correct, then gene and repeat family expansions commonly attributed to these prominent plant symbionts must have emerged later in the more derived AMF lineages, possibly to improve their adaptability to environmental and host change [11, 62, 63]. On the other hand, it is possible that the Paraglomerales have undergone drastic and species-specific genome reductive processes and the observation of over 200 *P. occultum* specific gene losses support this view. Either way, genome evidence from additional members of this order is needed to determine which scenario is more likely.

From an ecological perspective, it is intriguing to speculate that the gene family reduction we observed is linked to the reduced mycorrhizal capabilities recently reported for this order in comparison to AMF relatives [23, 24]. Alternatively, the smaller genome might be driven by selection for fast dividing life stages, adaptation to nitrogen limitation, or there may be due to unknown conditions where Paraglomerales are relatively more abundant (e.g. grow better than other Glomeromycota under high metal toxicity, or very high or very low pH, or salinity, etc.). Current analyses did not reveal evidence of gene expansions related to nitrate or metal metabolism and transport in *P. occultum* that could support this hypothesis, however, and data on the nuclear dynamics/division of early AMF lineages are currently non-existent [64].

### Conclusions

The present findings revealed that not all Glomeromycotina carry large and repetitive genomes. In fact, we show that early diverged members of this group can carry streamlined genomes with sizes and contents similar to those of other Mucoromycota subphyla. These reductive features can be explained by two contrasting hypotheses about the evolution of Glomeromycotina – i.e. late genome expansions vs lineage-specific adaptive reduction – and future studies should now focus on adding new annotated genome data from strains that belong to early lineages (including Paraglomerales) to address these conflicting views. Lastly, this work demonstrates that a complete, contaminant-free view of the genome content can be obtained from pot-cultured AMFs using a few nanograms of DNA, and without the need for expensive DNA/RNA extraction kits tailored to small amounts of starting material [5], or instruments dedicated to the isolation and sequencing of individual nuclei [54, 65].

## Supplementary Data

Supplementary material 1
