# Supplementary material for: Early branching arbuscular mycorrhizal fungus Paraglomus occultum carries a small and repeat-poor genome compared to relatives in the Glomeromycotina
Source: Microb Genom. 2022 Apr 22;8(4):000810. doi: 10.1099/mgen.0.000810 (PMC9453076; doi:10.1099/mgen.0.000810)
Supplement: Supplementary material 1 [file mgen-8-0810-s001.pdf]

Supplementary Tables:

Supplementary Table 1: Total secretory proteins predicted for this study from all genomes

Strain	Secreted gene count
Paraglomus Occultum	**209**
Geosiphon pyriformis	365
Gigaspora rosea v1.0	877
Mortierella elongata v2.0	810
*Mucor circinelloide*s v2.0	372
Phycomyces blakesleeanus v2.0	379
Rhizophagus cerebriforme v1.0	353
Rhizophagus diaphanus v1.0	378
Rhizophagus irregularis v2.0	526
Rhizopus microsporus v1.0	346
Diversispora epigea	483

Supplementary Table 2: Location of Meiosis specific genes from the assembled genome of *P. occultum*

Meiosis Specific genes	genome co-ordinates	***Paraglomus occultum*** **location**
DMC 1	scaffold_1752:1343-3169	FUN_002602-T1<br>FUN_002602

Hop2	scaffold_314:15016-16315	FUN_005394-T1<br>FUN_005394
MND1	scaffold_284:14461-16793	FUN_004908-T1<br>FUN_004908
MSH4	scaffold_374:8049-11503	FUN_006283-T1<br>FUN_006283
MSH5	scaffold_1742:1334-5016	FUN_002583-T1<br>FUN_002583
REC8	scaffold_367:6724-9812	FUN_006178-T1<br>FUN_006178
SPO11	scaffold_1592:3975-5367	FUN_002154-T1<br>FUN_002154

Supplementary Figures

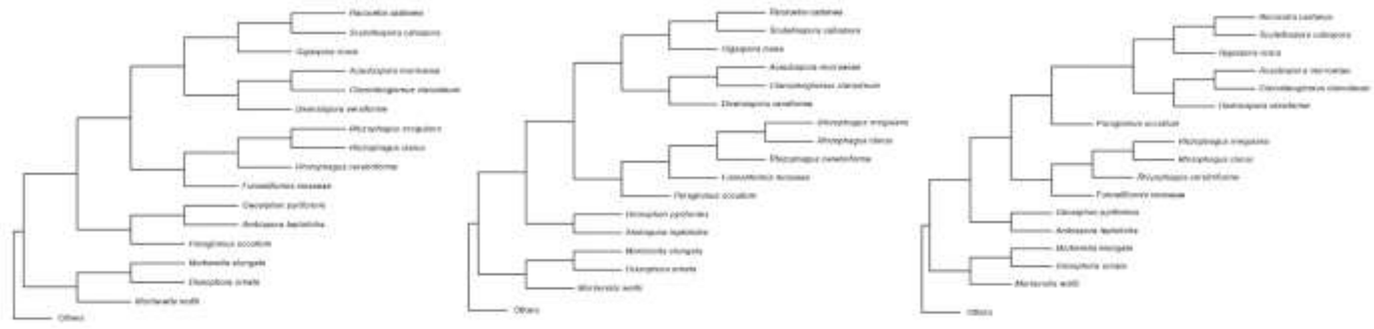

Alt_T1 Alt_T2 Alt_T3

Supplementary Figure 1: Alternative phylogenetic placements of *Paraglomus occultum* examined in the alternative topology tests. Three alternative topologies with the *Paraglomus* at the earliest diverged position within Glomeromycotina (Alt_T1), grouped with the *Rhizophagus* and *Funneliformis* clade (Alt_T2), and joined with the *Gigaspora* and *Acaulospora* clade (Alt_T3) were compared to the best tree produced in the present study given the genome-wide data set. The log-likelihoods of each of the four topologies were computed using the IQTREE package and the results including the Kishino-Hasegawa test, Shimodaira-Hasegawa test, expected likelihood weight, and approximately unbiased test were summarized in Supplemental File 4.

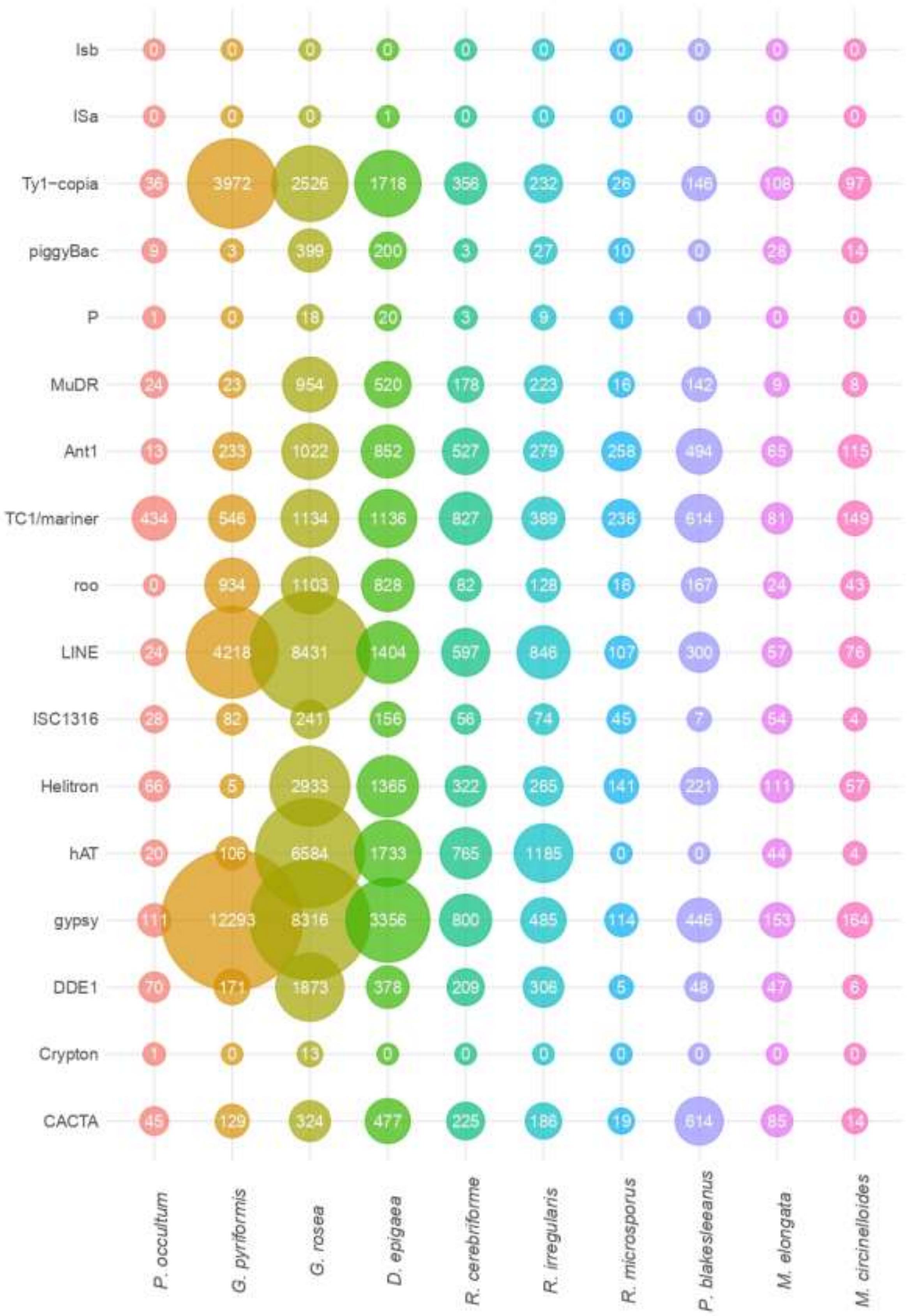
Isb 0 0 0 0 0 0 0 0 0 0
ISa 0 0 0 1 0 0 0 0 0 0
Ty1−copia 36 3972 2526 1718 358 232 26 146 108 97
piggyBac 9 3 399 200 3 27 10 0 28 14
P 1 0 18 20 3 9 1 1 0 0
MuDR 24 23 954 520 178 223 16 142 9 8
Ant1 13 233 1022 852 527 279 258 494 65 115
TC1/mariner 434 546 1134 1136 827 389 236 614 81 149
roo 0 934 1103 828 82 128 16 167 24 43
LINE 24 4218 8431 1404 597 846 107 300 57 76
ISC1316 28 82 241 156 56 74 45 7 54 4
Helitron 66 5 2933 1365 322 265 141 221 111 57
hAT 20 106 6584 1733 765 1185 0 0 44 4
gypsy 111 12293 8316 3356 800 485 114 446 153 164
DDE1 70 171 1873 378 209 306 5 48 47 6
Crypton 1 0 13 0 0 0 0 0 0 0
CACTA 45 129 324 477 225 186 19 614 85 14
P. occultum
G. pyriformis
G. rosea
D. epigaea
R. cerebriforme
R. irregularis
R. microsporus
P. blakesleeanus
M. elongata
M. circinelloides

Supplementary Figure 2: Bubble plot containing all transposable elements predicted in this study. X-axis shows the list of genomes used in this study and Y-axis shows the categories of TE elements identified.

Supplemental File 1: Identification of total cazyme using dbcan2 server

**Cazy family**	***Paraglomus occultum***	***Rhizhophagus irregularis***	***Rhizophagus cerebriforme***	***Gigaspora rosea***	***Diversispora epigaea***	***Geosiphon pyriformis***	***Rhizopus microsporus***	***Mortierella elongata***	***Mucor circinelloides***	***phycomyces blakesleeanus***
**Auxillary activity AA family**										
AA11	1	1	1	1	2	4	0	13	0	0
AA1	0	1	1	1	0	0	0	0	0	2
AA2	2	2	2	2	2	3	3	1	4	2
AA3	3	7	10	5	4	1	1	3	2	5
AA6	0	1	2	1	5	1	3	0	5	4
AA7	6	12	9	28	6	6	1	8	1	2
AA12	2	0	0	0	0	0	1	0	0	1
AA5	0	0	0	0	0	0	0	0	0	0
AA4	0	0	0	0	0	0	1	0	1	2
**Carbohydrate-Binding Modules Family**										
CBM21	1	1	1	1	1	1	5	2	6	4
CBM14	0	0	0	1	1	0	0	0	0	0
CBM42	0	0	0	0	1	0	0	0	0	0
CBM43	0	0	0	0	0	0	0	1	0	0
CBM19	0	0	0	0	0	0	1	0	0	0
CBM5	0	0	0	0	0	0	1	1	0	0
CBM13	0	0	0	0	0	0	0	2	0	0
**Carbohydrate esterases family**										
CE16	1	1	2	4	1	15	5	1	10	3
CE4	11	8	10	21	6	11	19	28	24	15
CE9	1	1	1	1	1	0	1	2	2	1

CE12	0	0	0	0	1	0	0	0	0	0
CE1	0	0	0	0	0	0	1	2	1	1
CE2	0	0	0	0	0	0	1	0	1	2
CE6	1	1	1	1	1	1	1	0	1	0
CE8	0	0	0	0	0	0	3	0	0	4
**Glycoside hydrolases family**										
GH125	0	2	1	0	0	3	2	0	2	2
GH15	1	1	1	1	1	1	4	2	4	4
GH18	2	1	1	11	3	3	12	11	15	8
GH20	2	1	2	2	1	3	4	6	4	4
GH24	0	5	5	6	0	0	0	1	0	0
GH31	3	2	3	3	4	2	3	3	5	3
GH35	1	2	2	2	1	1	1	1	1	1
GH36	0	0	0	0	0	0	1	0	2	2
GH37	1	1	1	2	2	2	3	2	5	4
GH38	1	1	1	1	1	1	2	1	2	2
GH47	4	5	6	6	4	5	9	6	7	7
GH63	1	1	1	2	1	1	1	2	0	0
GH9	1	2	2	6	1	0	3	2	4	2
GH23	0	0	2	0	0	0	0	0	0	0
GH25	0	0	0	1	0	0	0	0	0	0
GH81	0	0	0	1	0	1	1	1	1	1
GH85	1	0	0	2	1	1	0	1	1	0
GH92	0	0	0	10	0	0	0	0	0	0
GH27	1	0	0	1	0	1	0	1	0	0
GH17	1	0	0	0	1	4	2	5	3	1
GH25	0	0	0	0	3	0	0	1	0	0
GH152	0	0	0	0	0	1	4	0	4	5
GH72	0	0	0	0	0	0	1	2	1	2
GH28	0	0	0	0	0	0	5	1	2	9
GH45	0	0	0	0	0	0	4	0	0	1
GH3	0	0	0	0	0	0	5	1	5	3
GH46	0	0	0	0	0	0	2	0	1	1
GH89	0	0	0	0	0	0	0	3	0	0
GH8	0	0	0	0	0	0	1	1	2	1
GH134	0	0	0	0	0	0	4	0	3	0

GH2	0	0	0	0	0	0	1	0	0	0
GH65	0	0	0	0	0	0	1	0	0	0
GH76	0	0	0	0	0	0	1	0	0	0
GH95	0	0	0	0	0	0	2	0	0	0
GH29	0	0	0	0	0	0	0	0	2	0
GH32	0	0	0	0	0	0	0	0	0	1
GH51	0	0	0	0	0	0	0	0	0	1
**Glycosyl transferases family**										
GT15	2	3	4	9	3	2	12	3	15	12
GT1	1	26	11	36	3	0	9	2	7	1
GT20	3	3	3	3	2	2	6	4	8	7
GT21	1	1	1	1	0	1	1	2	1	1
GT22	2	4	4	4	4	4	4	4	4	4
GT24	1	1	1	1	2	1	1	1	1	1
GT28	1	1	1	0	0	0	0	0	0	0
GT31	1	3	2	4	1	2	3	3	3	1
GT33	1	1	1	1	1	1	1	1	1	1
GT34	2	6	8	2	2	2	1	2	2	2
GT35	1	1	1	1	1	1	2	1	2	0
GT39	3	4	4	7	3	3	6	9	8	7
GT3	2	2	2	1	1	1	2	4	2	2
GT48	1	1	1	0	1	1	2	2	3	3
GT49	3	3	3	3	3	2	8	7	8	5
GT4	3	4	4	5	4	2	5	3	6	5
GT50	1	1	1	1	1	1	1	1	1	1
GT57	1	2	2	2	2	2	2	2	2	2
GT58	0	1	1	1	1	1	1	1	1	1
GT59	0	1	1	1	1	1	2	1	1	2
GT5	1	2	2	1	1	0	2	1	2	2
GT66	1	1	1	1	1	1	1	1	1	1
GT69	0	1	0	0	0	0	0	0	0	1
GT76	0	1	1	1	1	1	1	1	1	1
GT8	1	2	1	2	3	3	3	1	3	3
GT25	0	0	1	20	7	0	0	0	0	0
GT10	5	0	0	11	0	0	1	10	2	1
GT23	0	0	0	2	0	0	0	4	0	0

GT47	0	0	0	4	0	0	1	0	1	1
GT10	0	0	0	0	4	0	0	0	0	0
GT62	0	0	0	0	0	1	2	2	6	2
GT64	0	0	0	0	0	0	1	0	2	1
GT71	0	0	0	0	0	0	3	7	4	3
GT77	0	0	0	0	0	0	3	0	3	1
GT17	0	0	0	0	0	0	0	1	0	1
**polysaccharide lyases family**										
PL38	2	5	4	3	1	3	2	3	2	2
PL14	0	0	0	0	0	0	1	0	1	0
PL8	0	0	0	0	0	0	1	0	1	1
PL29	0	0	0	0	0	0	0	2	0	0
**Subfamilies**										
**AA subfamily**										
AA1_1	0	4	2	1	4	1	0	0	0	0
AA1_2	1	4	3	13	3	0	2	3	4	2
AA5_1	8	8	12	4	2	3	2	4	1	1
AA3_2	0	0	1	13	2	0	2	4	1	0
AA3_1	0	0	0	1	2	1	0	0	0	0
AA3_4	0	0	0	4	0	0	0	0	0	0
AA1_3	0	0	0	0	2	0	0	0	0	0
**GH subfamily**										
GH13_1	1	1	1	1	0	3	1	0	0	0
GH13_25	1	1	1	1	1	1	1	1	1	1
GH13_8	1	1	1	1	1	1	1	2	1	1
GH5_12	1	2	2	2	2	1	2	5	2	2
GH5_27	0	1	1	0	0	0	0	2	1	0
GH5_7	1	1	1	2	1	1	1	0	2	1
GH16_19	3	0	1	2	3	5	3	5	3	2
GH16_10	0	0	0	5	0	0	0	0	0	0
GH30_5	0	0	0	13	0	0	0	0	0	0

GH5_9	1	0	0	2	0	5	2	3	2	2
GH16_1	0	0	0	0	0	0	2	1	2	0
GH16_23	0	0	0	0	0	0	2	0	2	1
GH16_4	0	0	0	0	0	0	1	1	1	1
GH13_5	0	0	0	0	0	0	0	1	0	0
GH16_21	0	0	0	0	0	0	0	4	0	0
GH5_50	0	0	0	0	0	0	0	0	0	1
**GT subfamily**										
GT2_Chitin_synth_1	3	6	6	18	2	2	4	6	6	7
GT2_Chitin_synth_2	8	10	10	9	9	5	22	12	20	18
GT2_Glycos_transf_2	2	2	3	2	3	2	2	2	2	2
GT2_Glyco_trans_2_3	0	1	1	1	1	1	2	0	4	0
GT2_Glyco_tranf_2_3	0	0	0	0	0	0	1	4	2	3
**PL subfamily**										
PL14_4	0	0	0	0	0	0	1	0	0	0
PL14_5	0	0	0	0	0	0	1	2	1	1
PL14_3	0	0	0	0	0	0	0	1	0	2
PL3_2	0	0	0	0	0	0	0	2	0	0
PL8_3	0	0	0	0	0	0	0	0	1	0
total cazyme	120	183	180	347	148	144	273	269	298	240

Supplemental File 2: Plant cell wall degrading enzymes across mucoromycotina genomes

**Family**	***Paraglomus occultum***	***R. irregularis***	***R. cerebriforme***	***G. rosea***	***D. epigaea***	***G. pyriformis***	***R. microsporus***	***M. elongata***	***M. circinelloides***	***p. blakesleeanus***
AA1	0	1	1	1	0	0	0	0	0	2

AA 1_2	1	4	3	13	3	0	2	3	4	2
AA 11	1	1	1	1	2	4	0	13	0	0
AA 12	2	0	0	0	0	0	1	0	0	1
AA 3_2	0	0	1	13	2	0	2	4	1	0
AA 3_4	0	0	0	4	0	0	0	0	0	0
AA 5	0	0	0	0	0	0	0	0	0	0
AA 5_1	8	8	12	4	2	3	2	4	1	1
AA 7	6	12	9	28	6	6	1	8	1	2
CB M1 3	0	0	0	0	0	0	0	2	0	0
CB M1 4	0	0	0	1	1	0	0	0	0	0
CB M1 9	0	0	0	0	0	0	1	0	0	0
CB M2 1	1	1	1	1	1	1	5	2	6	4
CB M4 3	0	0	0	0	0	0	0	1	0	0
CB M5	0	0	0	0	0	0	1	1	0	0
CE 16	1	1	2	4	1	15	5	1	10	3
CE 2	0	0	0	0	0	0	1	0	1	2
CE 4	11	8	10	21	6	11	19	28	24	15
CE 8	0	0	0	0	0	0	3	0	0	4
GH 13_ 1	1	1	1	1	0	3	1	0	0	0
GH 134	0	0	0	0	0	0	4	0	3	0
GH 15	1	1	1	1	1	1	4	2	4	4
GH 17	1	0	0	0	1	4	2	5	3	1

GH 18	2	1	1	11	3	3	12	11	15	8
GH 20	2	1	2	2	1	3	4	6	4	4
GH 24	0	5	5	6	0	0	0	1	0	0
GH 25	0	0	0	1	0	0	0	0	0	0
GH 25	0	0	0	0	3	0	0	1	0	0
GH 27	1	0	0	1	0	1	0	1	0	0
GH 28	0	0	0	0	0	0	5	1	2	9
GH 3	0	0	0	0	0	0	5	1	5	3
GH 30_5	0	0	0	13	0	0	0	0	0	0
GH 31	3	2	3	3	4	2	3	3	5	3
GH 35	1	2	2	2	1	1	1	1	1	1
GH 36	0	0	0	0	0	0	1	0	2	2
GH 37	1	1	1	2	2	2	3	2	5	4
GH 45	0	0	0	0	0	0	4	0	0	1
GH 46	0	0	0	0	0	0	2	0	1	1
GH 47	4	5	6	6	4	5	9	6	7	7
GH 5_27	0	1	1	0	0	0	0	2	1	0
GH 5_7	1	1	1	2	1	1	1	0	2	1
GH 5_9	1	0	0	2	0	5	2	3	2	2
GH 51	0	0	0	0	0	0	0	0	0	1
GH 63	1	1	1	2	1	1	1	2	0	0
GH 72	0	0	0	0	0	0	1	2	1	2
GH 76	0	0	0	0	0	0	1	0	0	0
GH 81	0	0	0	1	0	1	1	1	1	1

GH 9	1	2	2	6	1	0	3	2	4	2
GH 92	0	0	0	10	0	0	0	0	0	0
GH 95	0	0	0	0	0	0	2	0	0	0
PL 14	0	0	0	0	0	0	1	0	1	0
PL 14_3	0	0	0	0	0	0	0	1	0	2
PL 3_2	0	0	0	0	0	0	0	2	0	0

Supplemental File 3: Missing MRCA genes across glomeromycotina core genes. Absent genes are shown as NA

**Metabolism**	**Gene ID**	**Name**	**Description**	***P. occultum***	***G. pyriformis***	***R. irregularis***	***D. epigaea***	***R. cerebriforme***	***G. rosea***
thiamine metabolism/transport	YGR144W	THI4	Thiazole synthase	NA	NA	NA	NA	NA	NA
	YPL214C	THI6	Bifunctional enzyme with thiamine-phosphate pyrophosphorylase and 4-methyl-5-beta-hydroxyethyl thiazole kinase activities	NA	NA	NA	NA	NA	NA
	YLR237W	THI7	Plasma membrane transporter responsible for the uptake of thiamine	NA	NA	NA	NA	NA	NA

	YOL 055C	THI 20	Multifunctional protein with hydroxymethylpyrimidine phosphate (HMP-P) kinase and thiaminase activities	NA	NA	NA	NA	NA	NA
	YPL 258C	THI 21	Hydroxymethylpyrimidine phosphate kinase	NA	NA	NA	NA	NA	NA
	YPR 121 W	THI 22	hydroxymethylpyrimidine phosphate kinases	NA	NA	NA	NA	NA	NA
	YOR 192C	THI 72	Transporter of thiamine or related compound	NA	NA	NA	NA	NA	NA
	YOR 071C	NRT 1	High-affinity nicotinamide riboside transporter	NA	NA	NA	NA	NA	NA
**allantoine metabolism/transport**	YIR028W	DAL 4	Allantoin permease	NA	NA	NA	NA	NA	NA
**alcohol metabolism/fermentation**	YGL 256 W	ADH4	Alcohol dehydrogenase isoenzyme type IV	NA	NA	NA	NA	NA	NA
**uracil metabolism/transport**	YBL 042C	FUI1	High affinity uridine permease	NA	NA	NA	NA	NA	NA
	YBR 021 W	FUR 4	Uracil permease	NA	NA	NA	NA	NA	NA

**detoxification/stress response**	YER185W	PUG1	Plasma membrane protein with roles in the uptake of protoprophyrin IX and the efflux of heme	NA	NA	NA	NA	NA	NA
	YGR213C	RTA1	Protein involved in 7-ami-cholesterol resistance	NA	NA	NA	NA	NA	NA
	YGR234W	YHB1	Nitric oxide oxidoreductase	NA	NA	NA	NA	NA	NA
	YIL053W	RHR2	DL-glycerol-3-phosphatase	NA	NA	NA	NA	NA	NA
	YPR201W	ARR3	Arsenite transporter	NA	NA	NA	NA	NA	NA
	YGL196W	DSD1	D-serine dehydratase (aka D-serine ammonia-lyase)	NA	NA	NA	NA	NA	NA
	YHR044C	DOG1	2-deoxyglucose-6-phosphate phosphatase	NA	NA	NA	NA	NA	NA
	YHR043C	DOG2	2-deoxyglucose-6-phosphate phosphatase	NA	NA	NA	NA	NA	NA
**proteases/peptidases**	YHR132C	ECM14	Putative metalloprotease	NA	NA	NA	NA	NA	NA
**aromatic ami- acid metabolism**	YGL202W	ARO8	Aromatic ami-transferase I	NA	NA	NA	NA	NA	NA
	YHR137W	ARO9	Aromatic ami-transferase II	NA	NA	NA	NA	NA	NA

**channels/transporters**	YJL093C	TOK1	Outward-rectifier potassium channel	NA	NA	NA	NA	NA	NA
	YKL221W	MCH2	monocarboxylate permeases	NA	NA	NA	NA	NA	NA
**mating type/cell cycle/budding**	YIL140W	AXL2	Integral plasma membrane protein required for axial budding in haploid cells	NA	NA	NA	NA	NA	NA
**ER quality control**	YPL096W	PNG1	Conserved peptide N-glycanase required for deglycosylation of misfolded glycoproteins during proteasome-dependent degradation	NA	NA	NA	NA	NA	NA
	YBR015C	MNN2	Alpha-1,2-man-syltransferase	NA	NA	NA	NA	NA	NA
	YJL186W	MNN5	Alpha-1,2-man-syltransferase	NA	NA	NA	NA	NA	NA
**others**	YLL057C	JLP1	Fe(II)-dependent sulfonate/alpha-ketoglutarate dioxygenase, involved in sulfonate catabolism for use as a sulfur source	NA	NA	NA	NA	NA	NA

	YLR 047C	FRE 8	Iron/copper reductases, involved in iron homeostasis	NA	NA	NA	NA	NA	NA
	YLR 278C	YLR 278 C	Zinc-cluster protein	NA	NA	NA	NA	NA	NA
	YIL1 62W	SUC 2	Invertase (Glycosyl hydrolase)	NA	NA	NA	NA	NA	NA
	YKL 182 W	FAS 1	Fatty Acid Synthase	NA	NA	NA	NA	NA	NA
	YPL 231 W	FAS 2	Fatty Acid Synthase	NA	NA	NA	NA	NA	NA

Supplemental File 4: pathway analysis of transporters and Nitrogen metabolism using KASS server

**Pathway**	**Gene function**	**paraglomus**	**Rhizophagus cerebriforme**	**Rhizophagus irregularis**	**Gigaspora rosea**	**Geosiphon pyriformis**	**Diversispora epigea**	**Mortierella elongata**	**Mucor pirifo rmis**	**Phybl**	**Rhimi**
Nitrogen metabolism	Nrt (nitrogen transporter)	2	2	2	2	0	2	2	0	0	0
	Nr (nitrate reductase)	2	2	2	2	0	1	2	2	0	0
	NirBD (Nitrite reductase large subunit)	1	1	1	1	0	1	1	1	0	0
	1.7.1.15 nitrite reductase large subunit)	1	1	1	1	0	1	1	1	0	0
	4.2.1.1 (carbonic anhydrase)	1	1	1	1	1	1	1	1	1	1
	1.4.1.2(glutamate dehydrogenase)	1	1	1	1	1	1	1	1	1	1
	6.3.1.2(glutamine synthetase)	1	1	1	1	1	1	1	1	1	1
	1.4.1.14(glutamate synthatase)	1	1	1	1	1	1	1	1	1	1
	1.13.1216(nitronate monooxygenase)	0	1	1	0	0	1	0	1	1	1
	42.1.104 (cyanate lyase)	0	1	1	1	1	1	0	1	1	1

	1.4.1.3(glutamate dehydrogenase)	0	1	1	1	0	0	1	1	1	1
	1.4.1.4(glutamate dehydrogenase)	0	0	0	0	0	0	1	0	0	0
ABC transporters											
	ABCB1 (ATP-binding cassette, subfamily B (MDR/TAP), member 8)	1	1	1	1	1	0	0	1	1	0
	ABCA1	0	0	0	0	1	0	0	0	0	0
	ABCA2	0	0	0	0	0	0	1	0	0	0
	ABCA10	0	0	0	0	1	0	0	0	0	0
	ABCB1	0	0	0	0	0	1	1	0	0	1
	ABCB6(ATP-binding cassette, subfamily B (MDR/TAP), member 6)	1	1	1	1	1	1	1	1	1	1
	ABCB7(ATP-binding cassette, subfamily B (MDR/TAP), member 7)	1	1	1	1	1	1	1	1	1	1
	ABCB8(ATP-binding cassette, subfamily B (MDR/TAP), member 8)	1	0	0	0	0	0	0	1	1	1
	ABCB10	0	0	0	0	1	0	0	1	1	1
	Rv0194(ATP-binding cassette, subfamily B, multidrug efflux pump)	0	1	1	0	0	0	0	0	0	0
	MsbA(ATP-binding cassette, subfamily B, bacterial MsbA [EC:7.5.2.6])	0	0	0	1	1	1	0	1	0	0
	Atm1(ATP-binding cassette, subfamily B, heavy metal transporter)	1	0	0	0	0	0	0	0	0	0
	EfrA/B(ATP-binding cassette, subfamily B, multidrug efflux pump)	1	1	0	0	0	1	0	0	0	0
	ABCC1(ATP-binding cassette, subfamily C (CFTR/MRP), member 1 [EC:7.6.2.3])	1	1	1	1	1	1	1	1	1	1
	ABCC3	0	0	0	0	0	1	0	0	0	0
	ABCC4	0	0	0	0	0	0	1	0	0	0
	ABCC5	0	0	0	0	0	0	1	0	0	0
	PXA 1/2(ATP-binding cassette, subfamily D (ALD), peroxisomal long-chain fatty acid import protein)	1	1	1	1	1	1	0	1	0	0
	ABCD1	0	0	0	0	0	0	0	0	0	1

	ABCD2(ATP-binding cassette, subfamily D (ALD), member 2)	1	1	1	1	1	1	1	1	1	1
	ABCD3(ATP-binding cassette, subfamily D (ALD), member 3)	0	1	1	1	1	1	1	1	1	1
	ABCD4(ATP-binding cassette, subfamily D (ALD), member 4)	1	0	1	1	1	1	1	1	1	1
	ABCG2(ATP-binding cassette, subfamily G (WHITE), member 2)	1	1	1	1	1	1	1	1	1	1
	ABCA3(ATP-binding cassette, subfamily A (ABC1), member 3)	0	1	0	0	0	0	1	0	0	0
	PDR5(ATP-binding cassette, subfamily G (WHITE), member 2)	0	0	0	0	0	0	0	0	1	1
	SNQ2(ATP-binding cassette, subfamily G (WHITE), member 2, SNQ2)	0	0	0	0	0	0	1	1	1	1
Nucleocytoplasmic transport											
	ALADIN	0	1	1	1	1	1	1	1	1	1
	hCG1(nucleoporin-like protein 2)	1	1	1	1	1	1	0	0	0	1
	Gle1(nucleoporin GLE1)	1	1	1	1	1	0	1	1	0	1
	DDX19(ATP-dependent RNA helicase DDX19/DBP5)	1	1	1	1	1	1	1	1	1	1
	Rae 1(mRNA export factor)	1	1	1	1	1	1	1	1	1	1
	Nup98(nuclear pore complex protein Nup98-Nup96)	1	1	1	1	1	1	1	1	1	1
	Nup88(nuclear pore complex protein Nup88)	1	1	1	1	0	0	1	1	1	1
	Nup214	1	0	1	1	1	0	1	0	0	1
	RanGAP(Ran GTPase-activating protein 1)	1	1	1	1	1	1	1	1	1	1
	UBC9(ubiquitin-conjugating enzyme E2 I)	1	1	1	1	1	1	1	1	1	1
	SUMO(small ubiquitin-related modifier)	1	1	1	1	0	1	1	1	1	1
	Nup160(nuclear pore complex protein Nup160)	1	0	0	1	1	0	1	0	0	0
	Nup85(nuclear pore complex protein Nup85)	1	1	1	1	1	1	1	1	1	1
	Sec13(protein transport protein SEC13)	1	1	1	1	1	1	1	1	1	1
	Nup107(nuclear pore complex protein Nup107)	1	1	1	1	1	1	1	1	1	1
	Nup133(nuclear pore complex protein Nup133)	1	1	1	0	1	0	1	0	0	0
	Nup96(nuclear pore complex protein Nup96)	1	1	1	1	1	1	1	1	1	1

	Seh1(nucleoporin SEH1)	1	1	1	1	1	1	1	1	1	1
	Nup43(nuclear pore complex protein Nup43)	1	1	1	1	1	1	0	0	0	0
	Nup62(nuclear pore complex protein Nup62)	1	0	0	1	1	1	1	1	1	1
	Nup58/45(nuclear pore complex protein Nup58/45)	1	0	0	1	1	1	1	0	0	1
	Nup54(nuclear pore complex protein Nup54)	1	1	1	1	1	1	1	1	0	1
	Nup205(nuclear pore complex protein Nup205)	1	1	1	1	1	1	1	1	1	1
	Nup155(nuclear pore complex protein Nup155)	1	1	1	1	1	1	1	1	1	1
	Nup93(nuclear pore complex protein Nup93)	1	1	1	1	1	1	1	1	1	1
	pom152(nucleoporin POM152)	1	1	1	1	1	1	1	1	1	1
	pom33(transmembrane protein 33)	1	1	1	1	0	1	1	1	1	1
	IPOA(importin subunit alpha-5)	1	1	1	1	1	1	1	1	1	1
	IPOB(importin subunit beta-1)	1	1	1	1	1	1	1	1	1	1
	SPN1(snurportin-1)	1	1	1	1	1	1	1	1	1	1
	XPO(exportin-1)	1	1	1	1	1	1	1	1	1	1
	Ran(GTP-binding nuclear protein Ran)	1	1	1	1	1	1	1	1	1	1
	eEF1A(elongation factor 1-alpha)	1	1	1	1	1	1	1	1	1	1
	CBC(nuclear cap-binding protein subunit 1)	1	1	1	1	1	1	1	1	1	1
	NMD3(60S ribosomal export protein NMD3)	1	1	1	1	0	1	1	1	1	1
	Y14	0	1	1	1	1	1	1	1	1	1
	MAGOH(protein mago nashi)	1	1	1	1	1	1	1	1	1	1
	EIF4A3(elongation factor 1-alpha)	1	1	1	1	1	1	1	1	1	1
	ACIN1(apoptotic chromatin condensation inducer in the nucleus)	1	1	1	1	1	0	1	0	0	1
	SAP18(histone deacetylase complex subunit SAP18)	1	1	1	1	1	1	1	1	1	1
	RNPS1	0	1	1	1	1	1	1	0	0	0
	Pinin	1	1	1	0	1	1	0	0	1	1
	Ref/Aly(THO complex subunit 4)	1	1	1	1	1	1	1	1	1	1
	Upf1(regulator of nonsense transcripts 1)	0	1	1	1	0	1	1	1	1	1
	Upf2(regulator of nonsense transcripts 2)	1	1	1	1	0	1	1	1	1	1
	Upf3(regulator of nonsense transcripts 3)	1	1	1	1	1	1	1	0	1	1

	SRM160(serine/arginine repetitive matrix protein 1)	1	1	1	1	1	0	1	1	0	0
	UAP56	0	1	1	1	1	1	1	1	1	1
	PYM(partner of Y14 and mago)	1	0	0	1	0	1	1	0	0	0
	THOC1(THO complex subunit 1)	1	1	1	1	1	1	1	1	1	1
	THOC2(THO complex subunit 2)	1	1	1	1	1	1	1	0	0	1
	THOC5 (THO complex subunit 5)	1	1	1	1	1	1	1	0	1	1
	THOC7(THO complex subunit 7)	0	1	1	1	0	1	1	0	0	0
	TEX1(THO complex subunit 3)	1	1	1	1	1	1	1	1	1	1
SNARE interactions in vesicular transport											
	Stx1-4(syntaxin 1A)	2	2	2	2	2	2	2	2	2	2
	VAMP4(vesicle-associated membrane protein 4)	2	2	2	2	2	2	2	2	2	2
	STx16	2	2	2	2	2	2	2	2	2	2
	Vti 1(vesicle transport through interaction with t-SNAREs 1)	2	2	2	2	2	2	2	2	2	2
	VAMP7	1	1	1	1	1	1	1	1	1	1
	STx8	1	1	1	1	1	0	1	0	1	1
	STx5	2	2	2	2	2	2	2	2	2	2
	Bos1(golgi SNAP receptor complex member 2)	1	1	1	1	1	1	1	1	1	1
	Gos1(golgi SNAP receptor complex member 1)	1	1	1	1	1	1	1	1	1	1
	Sec22	2	2	2	2	2	2	2	2	2	2
	Ykt6(synaptobrevin homolog YKT6)	0	2	2	2	2	2	2	2	2	2
	Bet1(blocked early in transport 1)	0	1	0	0	1	1	1	1	0	1
	Stx18	1	1	1	1	1	1	1	1	1	1
	USE1(unconventional SNARE in the endoplasmic reticulum protein 1)	0	1	1	1	0	1	0	0	1	0
	Sec20	1	1	1	1	1	1	1	0	1	1

Supplemental File 5: Results of alternative tree topology test for phylogenetic tree

Tree	logL	deltaL	bp-RELL	p-KH	p-SH	c-ELW	p-AU
**Best tree**	-5959773	0	0.813 +	0.821 +	1 +	0.813 +	0.834 +
**Alt_T1**	-5959838	64.952	0.187 +	0.179 +	0.461 +	0.187 +	0.166 +

**Alt_T2**	-5965217	5444.4	0 -	0 -	0 -	0 -	4.17e-07 -
**Alt_T3**	-5965231	5458.1	0 -	0 -	0 -	0 -	3.73e-07 -

Legends for this table are provided below

deltaL: logL difference from the maximal logL in the set.
bp-RELL: bootstrap proportion using RELL method (Kishino et al. 1990).
p-KH: p-value of one sided Kishino-Hasegawa test (1989).
p-SH: p-value of Shimodaira-Hasegawa test (1999).
c-ELW: Expected Likelihood Weight (Strimmer & Rambaut 2002).
p-AU: p-value of approximately unbiased (AU) test (Shimodaira, 2002).
Plus signs denote the 95% confidence sets.
Minus signs denote significant exclusion.
All tests performed 10000 resamplings using the RELL method.

Supplemental File 6: File containing predicted top 100 Pfam family domains identified using Pfamscan

**Pfam domains**	***P. occultum***	***G. pyriformis***	***D. epigaea***	***R. irregularis***	***G. rosea***	***R. ceribriforme***	***R. microsporus***	***P. blakesleeanus***	***M. circinelloides***	***M. elongata***
Sel1	1066	2202	710	2732	1742	2610	212	228	248	930
WD40	412	406	390	437	430	432	520	530	533	1783
Pkinase_Tyr	349	308	2420	2226	1778	1031	3	3	4	21
LRR_6	331	12	120	69	3115	54	17	25	24	409
BTB	204	208	1079	823	515	540	12	35	7	18
Pkinase	198	147	403	527	445	370	224	232	243	212
TPR_8	164	35	249	70	493	82	24	23	32	28
TPR_1	143	12	0	20	271	21	9	6	8	12
RRM_1	125	129	0	141	145	144	146	151	131	133
TLD	112	141	862	468	389	261	5	5	5	5
Mito_carr	99	93	112	101	110	108	144	150	169	174
BACK	97	100	900	205	378	123	0	8	0	1
HMG_box	83	93	82	138	139	136	23	26	24	15
TPR_12	81	4	0	6	188	6	2	4	4	3

TPR_11	66	7	0	20	145	17	4	1	2	3
Helicase_C	62	67	84	89	94	82	77	73	80	91
TPR_16	61	20	0	19	45	20	10	10	15	19
TPR_2	59	10	100	11	162	11	4	6	3	5
Myb_DNA-binding	59	160	66	54	52	50	29	20	16	37
p450	49	66	104	205	369	115	34	56	42	32
DEAD	48	48	72	52	62	59	52	50	49	62
AAA	45	38	62	58	67	63	47	57	55	56
C2	44	33	46	41	32	35	38	37	36	41
ABC_tran	43	48	58	61	102	66	55	84	88	164
Methyltransf_25	41	54	61	83	103	74	39	29	27	13
F-box-like	38	57	80	138	196	158	52	146	88	162
zf-C2H2	37	52	64	124	75	121	122	148	159	159
CBS	33	24	25	27	25	26	38	36	38	32
Ank_2	33	28	23	34	45	35	40	28	34	33
Ras	31	27	33	33	72	30	54	56	64	42
Myb_DNA-bind_6	30	53	20	27	19	45	9	8	11	13
PPR_2	28	19	21	21	28	22	25	26	18	17
KH_1	28	25	0	50	25	34	21	19	24	29
PPR_3	27	31	30	22	19	16	21	18	23	19
PPR	27	19	24	16	18	21	25	22	24	24
DnaJ	27	25	27	32	34	36	32	32	38	30
AAA_lid_3	26	21	26	25	26	27	24	31	25	28
zf-CCHC	25	294	129	35	41	28	16	15	19	17
adh_short	25	35	25	49	64	40	20	23	35	47
TPR_19	24	7	0	6	17	4	13	10	12	8
Arm	24	24	25	24	25	22	30	38	36	28
Sugar_tr	23	13	13	10	24	11	29	29	56	21
SH3_1	23	23	0	28	24	31	37	25	32	32
HATPase_c	23	19	10	18	230	18	17	18	16	21
ABC_membrane	23	30	37	37	80	41	17	43	45	100
Response_reg	22	20	8	15	143	15	14	15	16	20

E1-E2_ATPase	22	13	0	27	25	22	27	30	34	39
LRR_8	21	14	11	16	23	12	24	25	23	24
EF-hand_7	21	12	19	29	24	19	26	27	35	31
UQ_con	20	21	23	55	32	35	23	20	22	26
PX	19	15	17	19	16	18	26	25	25	22
Cyt-b5	19	15	19	26	41	22	30	34	35	24
BTB_2	19	33	25	24	41	28	7	3	9	2
SPRY	18	8	18	90	183	52	11	10	11	7
RNA_pol_Rpb1_R	18	0	0	0	0	0	0	0	0	0
RabGAP-TBC	18	13	17	17	19	16	26	32	30	22
PUF	18	18	17	18	21	18	46	34	59	38
MFS_1	18	35	0	45	118	45	72	76	120	102
Metallophos	17	17	18	29	32	25	28	34	34	32
GATA	17	21	28	24	24	28	42	36	36	28
E1_DerP2_DerF2	17	3	0	37	13	21	3	3	4	14
zf-RING_2	16	16	31	22	24	20	16	20	29	33
ubiquitin	16	28	40	99	70	70	18	30	26	34
HisKA	16	15	4	12	167	11	7	8	7	14
AMP-binding	16	13	14	24	70	22	31	39	41	39
AAA-ATPase_like	16	2	32	12	6	21	0	0	0	2
SET	15	16	27	31	20	25	25	20	23	23
MMR_HSR1	15	14	0	27	31	21	14	14	15	20
Ion_trans	15	7	17	58	59	44	9	17	17	25
Zn_clus	14	17	40	20	99	31	37	39	40	37
SNF2_N	14	14	14	16	15	16	24	21	22	23
SH3_9	14	11	11	8	11	13	19	15	14	16
Proteasome	14	15	15	15	14	14	14	14	14	14
Prenyltrans	14	14	14	14	14	15	13	14	14	13

PH	14	19	18	23	24	24	35	32	32	25
Peptidase_S8	14	8	13	22	15	15	16	25	16	23
PCI	14	16	16	15	15	15	16	16	15	17
LSM	14	12	16	17	18	17	17	20	16	17
Cation_ATPase_C	14	6	10	16	18	13	13	15	17	20
zf-CCCH	13	16	17	17	20	17	18	13	14	12
RhoGAP	13	9	7	10	16	9	32	30	34	24
Polysacc_deac_1	13	11	0	9	22	11	23	20	32	32
PB1	13	11	0	10	15	15	7	10	6	11
LIM	13	13	7	12	11	12	29	34	31	25
HSP70	13	9	13	70	28	44	13	27	20	21
HLH	13	16	12	13	21	12	40	35	37	26
Cation_ATPase	13	8	10	17	15	13	15	20	20	20
Bromodomain	13	14	20	52	15	16	22	16	23	23
Aldedh	13	11	14	12	24	16	13	13	16	17
Actin	13	14	13	12	13	11	11	13	13	14
Abhydrolase_1	13	13	0	14	24	18	10	14	14	24
UCH	12	13	17	23	45	17	22	20	26	22
UBA	12	7	12	12	14	16	14	9	7	19
Pro_isomerase	12	10	11	10	12	10	17	14	15	14
O-FucT	12	8	9	16	14	20	5	6	6	16
Na_Ca_ex	12	12	8	25	35	27	16	14	18	14
Hydrolase	12	9	12	18	25	16	15	14	18	27
HA2	12	8	11	10	8	11	7	4	10	9
GTP_EFTU	12	14	13	14	22	18	19	21	17	19
G-patch	12	11	10	13	11	12	11	9	9	11

Supplemental File 7: presence of TE elements in Mucoromycota genomes used in this study and TE coverage

**TE type**	***Paraglomus occultum***	***Geosiphon pyriformis***	***Gigaspora rosea***	***Diversispora epigeae***	***Rhizophagus ceribriforme***	***Rhizophagus irregularis***	***Rhizopus microsporus***	***Phycomyces Blakesleenus***	***Mortierella Elongata***	***Mucor Circinelloides CBS 277.49***
cacta	45	129	324	477	225	186	19	614	85	14
Crypton	1	0	13	0	0	0	0	0	0	0
DDE_1	70	171	1873	378	209	306	5	48	47	6
gypsy	111	12293	8316	3356	800	485	114	446	153	164
hAT	20	106	6584	1733	765	1185	0	0	44	4
helitronORF	66	5	2933	1365	322	265	141	221	111	57
ISC1316	28	82	241	156	56	74	45	7	54	4
LINE	24	4218	8431	1404	597	846	107	300	57	76
ltr_Roo	0	934	1103	828	82	128	16	167	24	43
mariner	434	546	1134	1136	827	389	236	614	81	149
mariner_ant1	13	233	1022	852	527	279	258	494	65	115
MuDR_A_B	24	23	954	520	178	223	16	142	9	8
P_element	1	0	18	20	3	9	1	1	0	0
piggybac	9	3	399	200	3	27	10	0	28	14
TY1_Copia	36	3972	2526	1718	356	232	26	146	108	97
ISa	0	0	0	1	0	0	0	0	0	0
Isb	0	0	0	0	0	0	0	0	0	0
**Total TE counts**	882	22715	35871	14144	4950	4634	994	3200	866	751
**TE per MB**	0.324924	16.424177	20.99229	8.105148	2.344236	2.997579	0.396042	2.423628	0.360099	0.657066

Supplemental File 8: List of Ortholog families absent in *Paraglomus occultum* genome present in other AMF's and Mortierellomycotina spp

1,3-beta-glucanosyltransferase
4F5 protein family
50S ribosomal protein L22

50S ribosome-binding GTPase
5-formyltetrahydrofolate cyclo-ligase family
5'-nucleotidase, C-terminal domain
6-O-methylguanine DNA methyltransferase, DNA binding domain
ABC1 domain protein
ABC-2 type transporter
acetyltransferase, GNAT family
acyl-CoA-binding protein
adenosyl-L-methionine (AdoMet)-dependent tRNA (uracil-O(2)-)-methyltransferase (By similarity)
AhpC TSA family protein
AIG2-like family
Alpha-1,2-galactosyltransferase
Alpha-1,3-mannosyltransferase alg-2
alpha-1,6-mannosyltransferase
alpha-tubulin suppressor protein Aats1
Altered inheritance of mitochondria protein 24, mitochondrial
AMMECR1 family
aromatic ring-opening dioxygenase
Arsenical pump membrane protein
aryl-alcohol dehydrogenase
Assembly factor required for Rieske Fe-S protein RIP1 incorporation into the cytochrome b-c1 (CIII) complex. Functions as a chaperone, binding to this subunit within the mitochondrial matrix and stabilizing it prior to its translocation and insertion into the late CIII dimeric intermediate within the mitochondrial inner membrane. Modulates the mitochondrial matrix zinc pool (By similarity)
AT DNA binding protein
atp-dependent rna helicase
ATP-independent protease that degrades mitochondrial transit peptides after their cleavage. Also degrades other unstructured peptides (By similarity)
ATP-utilising chromatin assembly and remodelling N-terminal
auxin efflux carrier
Benzoate 4-monooxygenase cytochrome P450
Beta-galactosidase
Beta-ketoacyl synthase, N-terminal domain
Beta-lactamase
BolA-like protein
BRCA1 C Terminus (BRCT) domain
Cactus-binding C-terminus of cactin protein
Calcineurin-like phosphoesterase
Carboxypeptidase S1
cation binding
CDP-diacylglycerol-inositol 3-phosphatidyltransferase

Cellular morphogenesis protein
chloride channel
Choline kinase
cipC-like antibiotic response protein
CSL zinc finger
ctr copper transporter family protein
CutC family
Cx9C motif-containing protein 4, mitochondrial
cyclic nucleotide-binding
CYCLIN
Cysteine dioxygenase type I
cytidine deaminase
D-amino acid oxidase
DDT domain protein
deaminase
Demethylmenaquinone methyltransferase
Deoxycytidylate deaminase
deoxyuridine 5'-triphosphate nucleotidohydrolase
dienelactone hydrolase
D-isomer specific 2-hydroxyacid dehydrogenase, catalytic domain
DJ-1 PfpI family protein
DNA- helicase
DNL zinc finger domain-containing protein
Dolichol phosphate-mannose biosynthesis regulatory protein
Dolichyl-phosphate mannosyltransferase polypeptide 3
DUF1264 domain protein
DUF159 domain protein
DUF323 domain-containing protein
DUF431 domain-containing protein
DUF543 domain protein
DUF636 domain-containing protein
DUF726 domain protein
DUF907 domain-containing protein
DUF967 domain protein
E1-like activating enzyme involved in cytoplasm to vacuole transport (Cvt) and autophagy (By similarity)
EBP domain protein
ELMO/CED-12 family
Endo-1,3-beta-glucanase
endonuclease exonuclease phosphatase family protein

ER-derived vesicles protein erv14
erythromycin esterase
exonuclease
exosome-associated family
Filament-forming protein
flavodoxin and radical SAM domain protein
flotillin domain protein
Fn3-like domain (DUF1034)
Folate-sensitive fragile site protein Fra10Ac1
Found in mitochondrial proteome protein 29
Fumarylacetoacetase
FUN14 family
G2-specific protein kinase
GDSL lipase acylhydrolase family protein
Glutaryl-CoA dehydrogenase
glycerophosphoryl diester phosphodiesterase
Glycoside hydrolase family 18 protein
glycoside hydrolase family 24
glycoside hydrolase family 5 protein
GNAT family acetyltransferase Nat4
Got1/Sft2-like family
HAL protein kinase
HAMP domain
haspin protein kinase
helicase, member of the UBC2 RAD6 epistasis group. Functions with DNA repair protein RAD18 in error-free postreplication DNA repair. Involved in the maintenance of wild- type rates of instability of simple repetitive sequences such as poly(GT) repeats. Seems to be involved in maintaining a balance which acts in favor of error-prone non-homologous joining during DNA double-strand breaks repairs (By similarity)
HELICc
helix-loop-helix DNA-binding domain-containing protein
Hemerythrin HHE cation binding domain
HORMA domain
HPP family
HSF
Imidazoleglycerol-phosphate dehydratase
INSIG domain-containing protein
intron homing
Isoprenylcysteine carboxyl methyltransferase (ICMT) family
K09885 aquaporin rerated protein, other eukaryote

K13099 CD2 antigen cytoplasmic tail-binding protein 2
KOW motif domain protein
Lactoylglutathione lyase
La domain
Lipid binding protein
Lipid transfer protein
LisH
long-chain-fatty-acid-CoA ligase
L-PSP endoribonuclease family protein (Hmf1)
lysosomal cobalamin transporter. Required to export cobalamin from lysosomes allowing its conversion to cofactors (By similarity)
lysozyme
MAGE family
mating-type switching protein swi10
Mediates inactivation of the TORC1 complex in response to amino acid starvation. Required for meiotic nuclear division (By similarity)
meiosis-specific serine threonine-protein kinase mek1
Membrane magnesium transporter
metallo-protease
MIND kinetochore complex component Nnf1
mitogen-activated protein kinase MAF1
Myb-like DNA-binding domain
N-acylethanolamine amidohydrolase
Nadh-ubiquinone oxidoreductase b18 subunit
NIF domain protein
NmrA-like family
norsolorinic acid reductase
Nuclease domain-containing protein 1
nucleolin protein Nsr1
nucleolus protein
NUDIX domain
o-acetylhomoserine
oligoribonuclease
Oligosaccharyl transferase subunit
O-methyltransferase
Optic atrophy 3 protein (OPA3)
OPT oligopeptide transporter protein
Oxidoreductase-like protein, N-terminal
permease
Pfam:DUF2010

Pfam:DUF2044
Pfam:DUF889
Pfs, NACHT, and Ankyrin domain protein
polarized growth protein
Poly (ADP-ribose) glycohydrolase (PARG)
Potassium ion channel Yvc1
Potassium transporter
PPIC-type PPIASE domain
Prefoldin subunit
Pre-SET motif
Probable N6-adenine methyltransferase
propionyl-CoA carboxylase
Protoporphyrinogen oxidase
recombination hotspot-binding protein
RED-like protein N-terminal region
Rhodanese domain protein
RING-14 protein
S1/P1 Nuclease
SCAMP family
Scavenger mRNA decapping enzyme C-term binding
Sds3-like
Serum paraoxonase arylesterase family protein
Sgf11 (transcriptional regulation protein)
SIT4-Associating protein
SMR domain-containing protein
Snf1 kinase complex beta-subunit Gal83
SnoRNA binding protein
Spo7-like protein
SprT
SRR1
subunit Mnn9
Subunits I, II and III form the functional core of the enzyme complex
subunit Trm112
SUMO activating enzyme
Taurine catabolism dioxygenase TauD, TfdA family
TB2/DP1, HVA22 family
Tetracycline transporter
Thiopurine S-methyltransferase (TPMT)
Translin family

Tyrosyl-DNA phosphodiesterase
ubiquitin-40S ribosomal protein S31 fusion protein
ubiquitin-60S ribosomal protein L40 fusion protein
Ulp1 protease family, C-terminal catalytic domain
Universal stress protein family
Ureidoglycolate hydrolase
Vitamin-D-receptor interacting Mediator subunit 4
YCII-related domain
YdiU domain protein
Yippee zinc-binding protein
